# Liver and kidney concentrations of strontium, barium, cadmium, copper, zinc, manganese, chromium, antimony, selenium and lead in cats

**DOI:** 10.1186/1746-6148-10-163

**Published:** 2014-07-17

**Authors:** Nadine Paßlack, Barbara Mainzer, Monika Lahrssen-Wiederholt, Helmut Schafft, Richard Palavinskas, Angele Breithaupt, Jürgen Zentek

**Affiliations:** 1 Department of Veterinary Medicine, Institute of Animal Nutrition, Freie Universität Berlin, Königin-Luise-Str. 49, 14195 Berlin, Germany; 2 Federal Institute for Risk Assessment, Max-Dohrn-Str. 8-10, 10589 Berlin, Germany; 3 Department of Veterinary Medicine, Institute of Veterinary Pathology, Freie Universität Berlin, Robert-von-Ostertag-Str. 15, 14163 Berlin, Germany

**Keywords:** Cats, Elements, Liver, Renal cortex/medulla, Age, Sex, Chronic kidney disease

## Abstract

**Background:**

In order to provide new knowledge on the storage of strontium (Sr), barium (Ba), cadmium (Cd), copper (Cu), zinc (Zn), manganese (Mn), chromium (Cr), antimony (Sb), selenium (Se) and lead (Pb) in the feline organism, we measured the concentrations of these elements in the liver, renal cortex and renal medulla, evaluating also the impact of age, sex or the occurrence of a chronic kidney disease (CKD). The element concentrations in the tissues of 47 cats (22 male; 25 female; aged between 2 months and 18 years) were measured using inductively coupled plasma mass spectrometry.

**Results:**

Cu, Zn and Mn were the highest in the liver, followed by the renal cortex and the renal medulla. The Cd concentrations were lower in the renal medulla compared to the renal cortex and the liver, and Sr was higher in the renal medulla compared to the liver. The Se concentrations in the cortex of the kidneys were higher than in the medulla of the kidneys and in the liver. Higher Cd concentrations were measured in the renal cortex of female cats, while no further gender-related differences were observed. Except for Cr, Sb and Se, age-dependencies were detected for the storage of all elements. The occurrence of a CKD also affected the storage of the elements, with lower concentrations of Ba (renal medulla), Zn (renal cortex; renal medulla) and Mn (liver; renal medulla), but higher Cd concentrations (liver; renal cortex) in diseased cats.

**Conclusions:**

In conclusion, the present results provide new information on the accumulation of specific elements in the feline liver and kidneys, demonstrating a dependency on age and an impaired kidney function, but not on the sex of the animals.

## Background

The environmental pollution with the alkaline earth metals strontium (Sr) and barium (Ba), the heavy metals cadmium (Cd) and lead (Pb), and the metalloid antimony (Sb) could be important for their storage in the animals’ organism. Since plants incorporate these elements and provide the basic food resource for livestock, the element concentrations in specific indicator organs of the animals, such as liver or kidneys, can possibly reach high values. Besides a general health risk for the farm animals, the meat and organs of these animals are important as food for humans, but also for carnivorous animals like dogs and cats. Up to now, only few information is available on the storage of Sr, Ba, Cd, Pb and Sb in organs of cats. However, reference data may be important especially for the interpretation of pathological investigations. In this context, a differentiation between male and female animals seems to be indicated, since data from human studies sometimes suggest a sexual dimorphism for the storage of elements in organs [1,2]. Moreover, considering that older cats are often affected by chronic kidney diseases [3], it is interesting to evaluate if the accumulation of the mentioned elements is dependent on the feline kidney function. In particular, disorders in the renal excretory mechanisms may either increase or decrease the storage of elements in the organism. The present study aimed at measuring the concentrations of Sr, Ba, Cd, Pb and Sb in the liver and the renal cortex and medulla of cats. The cats showed a different age (2 months-18 years), allowing an evaluation of age-dependent variations in the storage of the elements. Moreover, male and female cats were considered, and also cats without or with a chronic kidney disease (CKD).

Besides the mentioned alkaline earth metals, heavy metals and metalloid, we further focused in the present study on the trace elements zinc (Zn), copper (Cu), chromium (Cr), manganese (Mn) and selenium (Se). Although the biological functions of these elements are well known [4,5], studies dealing with the concentrations of these elements in the feline organism are only scarce [6]. It is also critical, that relevant studies were often performed several decades ago. Considering that specific trace elements are more recently used in livestock to improve the health, but also performance parameters of the animals [7,8], it can be assumed that the organs of farm animals show a high variation in the concentrations of trace elements. Since animal by-products are often used in petfood [9,10], the daily intake of Zn, Cu, Mn, Cr and Se may also markedly differ between cats and become important as excess trace elements are stored in the feline organism. The present study therefore aimed at providing new data on the concentrations of Zn, Cu, Mn, Cr and Se in the liver, renal cortex and renal medulla of cats, also differentiating between the animal’s age, sex and the occurrence of a CKD.

All elements were measured using inductively coupled plasma mass spectrometry (ICP-MS). The results of this study should provide new reference data for future investigations in cats and give some practical indication on the relevance of the intake of specific elements in diseased cats.

## Results

Large individual differences were observed for all measured elements in the liver and kidneys of the cats (Table 1). The highest concentrations of Cu, Zn and Mn were detected in the liver, followed by the cortex and the medulla of the kidney (*P* < 0.05). The lowest Cd concentrations were measured in the renal medulla when compared to the renal cortex and liver (*P* < 0.05). The Sr concentration was lower in the liver compared to the renal medulla, and the Se concentration was highest in the cortex of the kidneys when compared to the medulla of the kidneys and the liver (*P* < 0.05). The concentrations of Ba, Cr, Sb and Pb did not differ among the tissues.

**Table 1:** Concentrations (μg/kg) of strontium (Sr), barium (Ba), cadmium (Cd), copper (Cu), zinc (Zn), manganese (Mn), chromium (Cr), antimony (Sb), selenium (Se) and lead (Pb) in the liver and kidneys of cats (n = 47)

	**Liver**	**Cortex of the kidney**	**Renal medulla**
**Sr**	71.9 (0.00-2699)^a^	118 (0.00-1027)^ab^	130 (0.00-926)^b^
**Ba**	136 (0.00-1659)	191 (0.00-2628)	168 (0.00-1049)
**Cd**	145 (7.30-803)^a^	157 (4.13-2920)^a^	99.3 (2.74-1349)^b^
**Cu**	21889 (2701-98689)^a^	2644 (1119-15656)^b^	1923 (485-5939)^c^
**Zn**	27844 (8825-89115)^a^	13536 (5839-35290)^b^	9275 (3778-18405)^c^
**Mn**	2950 (1311-13098)^a^	1078 (502-2592)^b^	691 (233-1692)^c^
**Cr**	214 (9.36-368)	186 (10.7-384)	168 (13.9-441)
**Sb**	132 (0.00-216)	127 (0.00-236)	126 (0.00-377)
**Se**	396 (73.3-1792)^a^	823 (68.4-3429)^b^	578 (90.1-3429)^a^
**Pb**	86.6 (0.82-442)	86.9 (0.39-289)	83.2 (0.00-335)

Median (Minimum-Maximum).

Differences (*P* < 0.05) within a row are marked with different superscript letters.

The concentrations of the measured elements in the liver and kidneys of the cats did only slightly differ depending on the sex of the animals (Table 2). In the cortex of the kidneys, lower concentrations of Cd were measured in male cats (117 (4.13-2920) μg/kg) compared to female cats (214 (7.50-872) μg/kg) (*P* < 0.05). When the cats were divided into different age groups (Additional file 1: Table S1), male cats between 1.5-4 years showed lower Cd concentrations in the renal cortex (98.7 (41.1-116) μg/kg) compared to the female cats (208 (141-347) μg/kg). In the same age group, the Cd concentrations in the renal medulla of male cats (46.9 (3.10-79.3) μg/kg) were lower compared to the Cd concentrations in the renal medulla of female cats (133 (104-179) μg/kg). Old male cats (12-18 years) showed higher Cd concentrations in the liver (503 (294-583) μg/kg) compared to female cats at the same age (165 (62.5-271) μg/kg).

**Table 2:** Concentrations (μg/kg) of strontium (Sr), barium (Ba), cadmium (Cd), copper (Cu), zinc (Zn), manganese (Mn), chromium (Cr), antimony (Sb) selenium (Se) and lead (Pb) in the liver and kidneys of cats, depending on the sex of the animals (♂: n = 22; ♀: n = 25)

	**Liver**		**Cortex of the kidney**		**Renal medulla**	
	**♂**	**♀**	**♂**	**♀**	**♂**	**♀**
**Sr**	117 (0.00-2699)	58.7 (0.00-316)	124 (11.3-1027)	118 (0.00-512)	117 (7.26-364)	130 (0.00-926)
**Ba**	169 (0.00-1659)	126 (0.00-236)	206 (0.00-2628)	155 (0.00-471)	174 (0.00-1049)	168 (0.00-405)
**Cd**	132 (7.30-583)	146 (31.9-803)	117 (4.13-2920)^a^	214 (7.50-872)^b^	77.6 (3.10-1349)	107 (2.74-525)
**Cu**	16900 (3750-98689)	26792 (2701-95029)	2903 (1255-15656)	2591 (1119-5651)	1963 (1094-5939)	1814 (485-4674)
**Zn**	26559 (8825-89115)	27844 (13379-69164)	15069 (5839-24389)	11818 (5872-35290)	10017 (5622-18405)	8050 (3778-17276)
**Mn**	3237 (1403-6162)	2714 (1311-13098)	1117 (511-2592)	1002 (502-2077)	645 (338-1651)	703 (233-1692)
**Cr**	229 (29.2-354)	214 (9.36-368)	214 (57.1-376)	151 (10.7-384)	170 (21.4-441)	142 (13.9-436)
**Sb**	132 (0.00-201)	132 (0.00-216)	130 (0.00-236)	127 (0.00-186)	131 (0.00-377)	125 (0.00-243)
**Se**	427 (73.3-1163)	396 (179-1792)	841 (68.4-2699)	823 (346-3429)	477 (123-2044)	587 (90.1-3429)
**Pb**	87.9 (2.40-442)	86.6 (0.82-337)	101 (1.35-289)	86.1 (0.39-267)	89.3 (0.44-300)	80.8 (0.00-335)

Median (Minimum-Maximum).

Group comparisons were separately calculated for each element and each tissue. Differences (*P* < 0.05) within a row are marked with different superscript letters.

When the cats were divided into different age groups, differences in the hepatic and renal element concentrations could be observed (Table 3). In the liver and renal cortex, the Cd concentrations were the highest in old cats between 12-18 years when compared to the younger cats. In contrast, the Mn concentrations in the liver (4222 (1311-13098) μg/kg) and the Cu concentrations in the cortex of the kidney (3315 (1637-15656) μg/kg) were higher in young cats up to 1 year compared to old cats between 12-18 years (2634 (1403-4248) μg Mn/kg and 2676 (1255-3167) μg Cu/kg) (*P* < 0.05). The Sr concentrations in the liver of young cats (0-1 years) were higher (165 (6.00-2699) μg/kg) than in the other age groups, however, this difference was only statistically significant when the young cats were compared with the cats between 4.5-7 years (46.8 (0.00-163) μg/kg). The Ba concentrations in the liver showed some variations among the age groups and differed (*P* < 0.05), when the cats between 1.5-4 years (214 (67.5-303) μg/kg) were compared with the cats between 4.5-7 years (116 (0.00-236) μg/kg) and 12-18 years (87.1 (0.00-215) μg/kg).

**Table 3:** **Concentrations (μg/kg) of strontium (Sr), barium (Ba), cadmium (Cd), copper (Cu), zinc (Zn), manganese (Mn), chromium (Cr), antimony (Sb), selenium (Se) and lead (Pb) in the liver and kidneys of cats, depending on the age of the animals**^**1**^

	**Age**	**Liver**	**Cortex of the kidney**	**Renal medulla**
**Sr**	**0-1**	165 (6.00-2699)^a^	157 (21.2-1027)^a^	178 (11.2-364)^a^
	**1.5-4**	65.3 (0.00-138)^ab^	72.8 (48.6-132)^b^	69.1 (7.26-153)^b^
	**4.5-7**	46.8 (0.00-163)^b^	82.6 (0.00-251)^ab^	130 (0.00-625)^ab^
	**8-11**	112 (29.6-228)^ab^	139 (42.1-728)^a^	128 (42.7-207)^ab^
	**12-18**	92.5 (12.9-166)^ab^	144 (37.2-512)^ab^	157 (65.7-926)^a^
**Ba**	**0-1**	132 (0.00-662)^ab^	154 (0.00-258)^a^	202 (0.00-1049)^ab^
	**1.5-4**	214 (67.5-303)^a^	244 (207-387)^b^	221 (110-262)^a^
	**4.5-7**	116 (0.00-236)^b^	151 (0.00-258)^a^	118 (0.00-245)^ab^
	**8-11**	147 (0.00-1659)^ab^	205 (69.0-2628)^ab^	106 (0.00-960)^ab^
	**12-18**	87.1 (0.00-215)^b^	126 (0.00-471)^a^	84.4 (0.00-405)^b^
**Cd**	**0-1**	123 (7.30-264)^a^	116 (4.13-231)^a^	109 (2.74-225)
	**1.5-4**	138 (26.5-226)^a^	108 (41.1-347)^a^	77.6 (3.10-179)
	**4.5-7**	109 (50.4-803)^a^	144 (51.8-467)^a^	72.5 (3.98-525)
	**8-11**	192 (86.4-504)^ab^	178 (106-444)^ab^	88.9 (38.0-160)
	**12-18**	256 (62.5-583)^b^	353 (151-2920)^b^	137 (43.9-1349)
**Cu**	**0-1**	21507 (4237-98689)	3315 (1637-15656)^a^	2354 (1253-5939)
	**1.5-4**	13803 (3750-86070)	2454 (1814-3291)^ab^	2075 (1094-2792)
	**4.5-7**	29308 (2701-72700)	2458 (1119-5651)^ab^	1781 (485-2761)
	**8-11**	38696 (8876-91443)	3040 (1731-3598)^ab^	1648 (1217-2594)
	**12-18**	21768 (5236-48483)	2676 (1255-3167)^b^	1778 (895-2531)
**Zn**	**0-1**	40654 (16608-69164)	16145 (8280-33896)	13602 (5005-18405)^a^
	**1.5-4**	26115 (14353-35020)	13666 (10672-17993)	10375 (5655-15193)^ab^
	**4.5-7**	20174 (14975-41960)	9710 (8431-35290)	6924 (4687-15712)^b^
	**8-11**	32389 (15576-89115)	15747 (11051-19305)	9275 (6036-11938)^b^
	**12-18**	31502 (8825-60234)	11713 (5839-27943)	8741 (3778-14762)^b^
**Mn**	**0-1**	4222 (1311-13098)^a^	1547 (786-2592)^a^	920 (348-1692)^ab^
	**1.5-4**	3054 (2111-4144)^ab^	1240 (836-1969)^ab^	772 (338-1651)^a^
	**4.5-7**	2558 (1485-7073)^ab^	936 (502-1654)^b^	697 (233-916)^ab^
	**8-11**	2700 (1767-5052)^ab^	1086 (738-1312)^ab^	581 (417-733)^b^
	**12-18**	2634 (1403-4248)^b^	1031 (511-1452)^b^	587 (358-869)^ab^
**Cr**	**0-1**	287 (9.36-368)	270 (10.7-327)	275 (16.0-441)
	**1.5-4**	134 (55.0-345)	127 (50.7-291)	176 (53.3-337)
	**4.5-7**	196 (13.6-337)	139 (32.4-314)	135 (13.9-313)
	**8-11**	215 (60.8-354)	222 (71.5-376)	135 (21.4-296)
	**12-18**	269 (33.8-347)	205 (123-384)	255 (90.0-436)
**Sb**	**0-1**	185 (0.00-191)	180 (0.00-236)	182 (0.00-377)
	**1.5-4**	20.7 (0.00-191)	11.6 (0.00-180)	11.3 (0.00-180)
	**4.5-7**	129 (0.00-201)	116 (0.00-186)	115 (0.00-217)
	**8-11**	28.9 (0.00-194)	11.8 (0.00-187)	11.8 (0.00-184)
	**12-18**	136 (0.00-216)	131 (0.00-186)	127 (0.00-189)
**Se**	**0-1**	638 (73.3-844)	1168 (257-1898)	822 (123-1316)
	**1.5-4**	372 (215-1792)	726 (68.4-3429)	458 (231-3429)
	**4.5-7**	287 (179-1167)	651 (405-1687)	303 (90.1-1912)
	**8-11**	531 (258-693)	938 (408-2145)	463 (265-1315)
	**12-18**	660 (174-1421)	1473 (321-2699)	912 (223-2044)
**Pb**	**0-1**	230 (0.82-442)^ac^	214 (1.63-289)^ae^	219 (0.63-300)^a^
	**1.5-4**	39.3 (2.40-215)^bc^	24.2 (1.35-213)^abc^	8.85 (0.44-213)^b^
	**4.5-7**	70.5 (2.40-212)^b^	84.2 (0.39-229)^bd^	81.8 (0.00-329)^bc^
	**8-11**	140 (2.56-337)^abc^	137 (28.5-214)^de^	62.4 (2.47-202)^bc^
	**12-18**	154 (1.51-294)^a^	146 (27.7-267)^de^	141 (19.9-335)^ac^

Median (Minimum-Maximum).

^1^n = 10 (0-1 years), n = 8 (1.5-4 years), n = 12 (4.5-7 years), n = 7 (8-11 years), n = 10 (12-18 years).

Group comparisons were separately calculated for each element and each tissue. Differences (*P* < 0.05) within a column are marked with different superscript letters.

In the cortex of the kidneys, higher concentrations of Sr were measured in very young cats up to 1 year (157 (21.2-1027) μg/kg) and older cats between 8-11 years (139 (42.1-728) μg/kg), compared to cats between 1.5-4 years (72.8 (48.6-132) μg/kg). In contrast, these cats between 1.5-4 years showed higher concentrations of Ba in the renal cortex when compared to the very young cats (0-1 years) and the older cats (4.5-7 and 12-18 years) (*P* < 0.05).

Comparable to the results in the liver of the cats, the Mn concentrations in the renal cortex were higher in cats up to 1 year than in older cats (4.5-7 years and 12-18 years) (*P* < 0.05).

The measurement of the elements in the renal medulla demonstrated higher Zn concentrations in the very young cats between 0-1 years (13602 (5005-18405) μg/kg) compared to the older cats between 4.5-18 years (medians between 6924-9275 μg/kg) (*P* < 0.05).

Similar to the results in the cortex of the kidney, lower concentrations of Sr were measured in the renal medulla of cats between 1.5-4 years when compared to very young cats (0-1 year) and older cats (12-18 years) (*P* < 0.05).

The detection of Ba and Mn in the medulla of the kidneys revealed only single group differences when the cats between 1.5-4 years were compared with the cats between 12-18 years (Ba) or with the cats between 8-11 years (Mn).

The Pb concentrations in the liver and kidneys of the cats were high in very young cats, decreased in the middle-aged cats and subsequently increased in older cats (*P* < 0.05). No age-dependencies were detected for the concentrations of Cr, Sb and Se in the tissues of the cats.

The concentrations of the measured elements in the liver and kidneys of the cats with or without a CKD are summarized in Table 4. Cats with a CKD showed higher concentrations of Cd in the liver and renal cortex compared to cats without an impaired kidney function (*P* < 0.05). In contrast, the concentrations of Ba (renal medulla), Zn (renal cortex and medulla), and Mn (liver, renal medulla) were lower in cats with a CKD compared to cats without a CKD (*P* < 0.05).

**Table 4:** Concentrations (μg/kg) of strontium (Sr), barium (Ba), cadmium (Cd), copper (Cu), zinc (Zn), manganese (Mn), chromium (Cr), antimony (Sb), selenium (Se) and lead (Pb) in the liver and kidneys of cats without (w/o) or with a chronic kidney disease (CKD) (w/o: n = 25; CKD: n = 22)

	**Liver**		**Cortex of the kidney**		**Renal medulla**	
	**w/o**	**CKD**	**w/o**	**CKD**	**w/o**	**CKD**
**Sr**	71.9 (0.00-2699)	74.9 (4.33-164)	129 (0.00-1027)	118 (11.3-512)	84.0 (0.00-926)	130 (28.8-625)
**Ba**	192 (0.00-1659)	95.7 (0.00-248)	210 (0.00-2628)	149 (0.00-471)	205 (0.00-1049)^a^	80.8 (0.00-251)^b^
**Cd**	126 (7.30-503)^a^	163 (50.4-803)^b^	125 (4.13-2920)^a^	251 (80.0-872)^b^	99.3 (2.74-1349)	103 (42.9-525)
**Cu**	18009 (3750-98689)	25214 (2701-91443)	2746 (1119-15656)	2618 (1255-5651)	1923 (485-5939)	1941 (1093-2761)
**Zn**	27610 (14353-89115)	29018 (8825-48039)	14376 (5872-33896)^a^	11355 (5839-35290)^b^	10486 (4911-18405)^a^	8036 (3778-15712)^b^
**Mn**	3472 (1311-13098)^a^	2662 (1403-5060)^b^	1126 (738-2592)	1024 (502-1654)	735 (348-1692)^a^	624 (233-916)^b^
**Cr**	214 (9.36-368)	213 (31.5-354)	170 (10.7-384)	195 (32.4-339)	168 (16.0-441)	153 (13.9-368)
**Sb**	132 (0.00-194)	127 (0.00-216)	128 (0.00-236)	121 (0.00-186)	134 (0.00-377)	121 (0.00-217)
**Se**	396 (73.3-1792)	402 (174-1421)	900 (68.4-3429)	803 (321-2422)	634 (90.1-3429)	463 (223-1844)
**Pb**	86.6 (0.82-442)	86.6 (1.51-337)	93.5 (0.39-289)	86.9 (1.31-267)	95.3 (0.00-335)	82.1 (1.83-270)

Median (Minimum-Maximum).

Group comparisons were separately calculated for each element and each tissue. Differences (*P* < 0.05) within a row are marked with different superscript letters.

## Discussion

Up to now, only few data are available for the concentrations of Sr, Ba, Cd, Cu, Zn, Mn, Cr, Sb, Se and Pb in the liver and kidney of cats. The present study can extend the data pool, particularly distinguishing between the age and sex of the animals. Moreover, since a CKD often appears in older cats, this aspect was also considered by evaluating the storage of the elements in the feline organism depending on the occurrence of an impaired kidney function. In particular, it was assumed that disorders in the renal excretory mechanisms may increase or decrease the accumulation of the elements in the organs of cats. For the interpretation of the results, however, it should be considered that all animals were affected by different pathologies, while tissue samples from healthy cats were not collected for ethical reasons.

As a main observation, the highest concentrations of Zn, Cu and Mn were detected in the liver of the cats, followed by the renal cortex and the renal medulla. Since the liver is a storage organ for Cu [11], the hepatic Cu concentrations reflect the amounts of Cu in feed. Although the present study design did not allow an evaluation of the feeding regime of the cats, the high individual variations of the measured data suggest variations in the daily Cu intake. In general, the measured hepatic Cu concentrations (21889 (2701-98689) μg/kg fresh tissue) were comparable to previously reported data of Doong et al. [12] (9.4-57 μg/g fresh tissue in kittens and 53-68 μg/g fresh tissue in lactating queens), Haynes and Wade [13] (148-180 μg/g dry matter (DM)), Meertens et al. [14] (26-174 μg/g DM) and Andreani et al. [6] (38.1 ± 28.76 μg/g fresh tissue) in cats. An excessive Cu storage in the liver (4074 μg/g DM and 4170 μg/g DM) has been reported to be associated with hepatopathies in cats [13,14]. The measured Cu concentrations in the renal cortex (2644 (1119-15656) μg/kg fresh tissue) and renal medulla (1923 (485-5939) μg/kg fresh tissue) of the cats were also in accordance with data of Doong et al. [12] (1.7-2.7 μg/g fresh tissue in kittens and 2.3-2.7 μg/g fresh tissue in lactating queens).

The Cu concentrations in the liver and kidney of the cats did not differ depending on the sex of the animals. This is in contrast to findings in humans [1], where women showed higher Cu concentrations in the liver compared to men. These authors also demonstrated that the Cu concentrations in the liver, kidneys and rips decreased with increasing age. This is in accordance with the present results on the Cu concentrations in the renal cortex, where higher amounts were observed in young cats up to 1 year compared to old cats between 12-18 years, but in contrast to another study in cats, where no age-dependencies were observed for the renal and hepatic Cu accumulation [6].

The present observation that higher Zn concentrations were measured in the feline liver compared to the kidneys is supported by another study in cats [15], where female cats showed hepatic Zn concentrations of 109 mg/kg DM and male cats of 102 mg/kg DM, whilst 65 mg Zn/kg DM were measured in the kidneys. On the analogy of the present results, the authors could also find no gender-specific differences in the Zn concentrations of the feline liver and kidney. The measured Zn concentrations in the liver (27844 (8825-89115) μg/kg) and renal cortex (13536 (5839-35290) μg/kg) are in accordance with data of Doong et al. [12] in kittens (24.3-51 μg Zn/g liver and 12.7-19.8 μg Zn/g kidney) and lactating queens (27-34 μg Zn/g liver and 15.4-16.6 μg/g kidney).

The present data demonstrated an age-dependency for the Zn concentrations in the renal medulla, with the highest concentrations in young cats up to 1 year and lower concentrations in older cats. Although no significant differences could be detected in the renal cortex and liver of the cats, the findings in the renal medulla also applied to the other tissues, and are supported by data from humans, where children up to 1 year accumulated more Zn in the rips compared to older persons [16]. However, another study could not demonstrate age-dependencies for the storage of Zn in the liver and kidneys of cats [6].

The Mn concentrations in the liver and kidneys of the cats were independent of the sex of the animals, but showed age-related variations. In general, higher concentrations were measured in younger animals compared to older animals. This finding is in accordance with data from humans, where the Mn concentrations in the kidneys and rips decreased with age [16], however, other authors could not demonstrate an age effect on the Mn storage in organs of humans [17]. In this context, only one study is available in cats, but the authors also found higher Mn concentrations in the liver and bones of kittens compared to adult cats [18]. Another study measured the Mn concentrations in the liver and kidneys of kittens (1.7-4.5 μg/g liver and 0.5-3.3 μg/g kidney) and lactating queens (2.6-3.8 μg/g liver and 1.8-2.0 μg/g kidney), while these age-groups were not statistically compared [12].

Comparable Cd concentrations were found in the liver and renal cortex of the cats, while lower amounts were detected in the renal medulla. Moreover, female cats showed higher Cd concentrations in the cortex of the kidneys compared to male cats. This is in accordance with data from humans, where women showed higher Cd concentrations in the liver and kidneys compared to men [2]. The Cd concentrations in the liver and renal cortex were also dependent on the age of the cats, with lower concentrations in animals between 0-7 years compared to cats between 12-18 years, which is in accordance with another study in cats [6]. In humans, an increase of Cd was observed up to an age of 50 years, followed by a decrease of the Cd concentrations at an older age [16].

With regard to the measured Cd, but also Zn and Cu concentrations in the liver and kidneys of the cats, the role of metallothioneins in the homeostasis and storage of these metals should not go unmentioned. Metallothioneins are low molecule mass proteins with a high affinity to specific metal ions [19]. Therefore, metallothioneins are not only important for detoxification of heavy metal ions (e. g. Cd), but also for the homeostasis of Cu and Zn ions [19]. In particular, metallothioneins have been assumed to provide a pool of Zn, where Zn can be released when required [20] and to be important for the storage of Cu [19]. The present study design did not include the measurement of metallothioneins, and to our best knowledge, only one study has been performed to isolate Cd-, Zn- and Cu-metallothioneins in tissues of cats [6]. Overall, future studies are required to evaluate the potential of metallothioneins as markers for the exposure of cats to specific elements.

The Sr concentrations in the liver were lower when compared to the amounts in the renal medulla, while no difference was observed when the Sr concentrations in the cortex of the kidneys were compared with the concentrations in the other tissues. However, considering the broad range of the data, large individual differences can be assumed for the storage of Sr. While no gender-dependent differences were demonstrated for the Sr concentrations in the liver and kidney of the cats, clear age-related variations were found. In all tissues, the highest Sr concentrations were found in kittens up to 1 year. In the renal cortex and medulla, the lowest Sr concentrations were measured in cats between 1.5-4 years, followed by an increase at an older age. Similar results were detected in the liver, with the lowest Sr concentrations in cats between 4.5-7 years and a numerical increase in the following years of age. The present observations are in accordance with findings in humans, where newborns and children up to 9 years showed markedly higher concentrations of Sr in the liver and kidney compared to older persons, but humans over 50 years showed an increased accumulation of Sr in the kidneys [21]. An explanation for the marked higher Sr concentrations in the tissues of kittens and children can be found in the observation that milk increases the intestinal absorption of Sr [22]. The increased Sr accumulation at an older age, as observed in the cats of the present study, but also in humans [21], remains unclear up to now. In general, diet behavior at an older age is not similar between cats and humans. However, long-term ingestion of organs from meat producing animals or environmental pollution might be factors for the observed age-related accumulation of Sr in the organism.

Higher Se concentrations were found in the renal cortex when compared to the renal medulla and the liver of the cats. Up to now, only few studies have evaluated the Se status and metabolism in cats, however, such investigations are practically relevant, since high variations in dietary Se supply have been reported [23,24]. In general, the bioavailability of Se in canned and dry petfood has been demonstrated to be low [25-27], while the absorption of Se in the intestine is not only influenced by its dietary concentration, but also by its chemical form and its interaction with other diet-related factors like heavy metal concentrations or heating processes [28,29]. Considering these factors that might impact the intestinal absorption of Se in cats, variations in its storage could be assumed. However, the data of the present study only demonstrated a different storage among the tissues, while no gender-, age- or health-related differences were observed. This observation might be explained by the ability of cats to excrete excess dietary Se by the kidneys instead of a hepatic storage, which has been recently demonstrated [30]. In this study [30], hepatic Se concentrations of 1.37 ± 0.05 μg/g DM were measured, when cats received a moist control diet, which is in accordance with the present data. When Na_2_SeO_3_ or organic Se was added to the control diet, the hepatic Se concentrations (4.24 ± 0.59 μg/g DM and 4.79 ± 0.37 μg/g DM), but especially the renal Se excretion increased. Another study [31] found marked higher Se concentrations in the liver (mean: 0.69-21.4 μg/g, fresh basis) and kidneys (mean: 1.54-4.15 μg/g, fresh basis) of cats fed different experimental diets when the data are compared with the results of the present study.

The measurement of Pb in the liver and kidneys of the cats demonstrated similar concentrations among the tissues, but an age-dependency for its storage. In all tissues, highest Pb concentrations were measured in very young cats up to 1 year, lowest concentrations in middle-aged cats and a subsequent increase of the hepatic and renal Pb concentrations was observed at an older age of the cats. These findings are in accordance with data on the hepatic Pb accumulation in dogs [32], however, the authors of this study could not detect an age-dependency for the storage of Pb in the canine kidneys. In another study, no age-dependency was observed for the Pb concentrations in the liver and kidneys of dogs, but dogs fed a commercial diet showed significantly higher hepatic Pb concentrations compared to dogs fed home-made diets or a mixture of home-made diet and commercial diet [33]. To our best knowledge, no comparable study has been performed in cats up to now. However, further investigations would be practically relevant, since canned diets for cats have previously been identified to contain high amounts of Pb, where organ meats were assumed to be the main Pb source [34]. In this context, the present data can provide important reference data for the Pb storage in cats, however, a diet-related differentiation would be interesting for further studies.

The Ba, Cr and Sb concentrations did not differ between the liver and kidneys of the cats, and also no gender-specific differences could be detected. With the exception of Ba, no age-dependencies were observed, with the highest (*P* < 0.05) Ba concentrations in the liver and kidney of cats between 1.5-4 years. Since no further data are available for cats and only few results exist from human studies or investigations with other animals, the measured concentrations of Ba, Cr and Sb in the feline liver and kidney can be considered as important reference values, which will enhance the data pool.

The occurrence of a CKD partially affected the concentrations of the measured elements in the renal cortex and medulla, but also in the liver of the cats. The Cd concentrations in the liver and renal cortex were higher in cats with a CKD compared to healthy cats. Moreover, lower concentrations of Zn were detected in the renal cortex and medulla of cats with a CKD, which is in contrast to results of a previous study, where lower amounts of Zn were measured in the cerebrum, kidneys and liver of healthy cats compared to diseased cats [15]. However, in this study [15], not exclusively kidney diseases were considered, and especially cats with feline infectious peritonitis showed higher Zn concentrations in the tissues compared to healthy cats.

Lower Mn concentrations were measured in the liver and renal medulla of cats with a CKD compared to cats without an impaired kidney function. This observation is in accordance with a previous study, where lower Mn concentrations were found in the liver, kidneys, cardiac muscle, cerebrum, rips, testes, lung, and whole blood of humans with a CKD compared to healthy persons [35]. However, up to now it remains unclear, why less Mn is stored in diseased tissue. In a study with cats, a higher Mn accumulation was found in the liver of diseased cats, while no effect of the health status was observed for the Mn concentrations in the kidneys of the animals [15].

## Conclusions

Since only few data are available on the concentrations of Sr, Ba, Cd, Cu, Zn, Mn, Cr, Sb, Se and Pb in the feline organs, the present results can be considered as important reference data for diagnostics and future investigations. The sex of the animals had only minor impact on the concentrations of the elements in the liver and kidney of the cats, but differences depending on the age of the animals were observed. Interestingly, the occurrence of a CKD affected the storage of Ba, Cd, Zn and Mn, indicating that the renal excretory mechanisms could be a considerable factor for the storage of the elements in the feline organism.

## Methods

### Tissue samples and measurement of Sr, Ba, Cd, Cu, Zn, Mn, Cr, Sb, Se and Pb

The liver and kidney samples were collected during routine post-mortem examinations of euthanized cats in the Institute of Veterinary Pathology, Department of Veterinary Medicine, Freie Universität Berlin. The cats were euthanized by the treating veterinarian, and none of the authors participated. The euthanasia was performed based only on the clinical diagnosis and not for the reason of tissue sampling or other research. The euthanasia was carried out according to the German Tierschutzgesetz (animal welfare act), while details on the drugs are unspecified. In total, 47 cats (22 male, 25 female) were included in this study, aged between 2 months and 18 years. The pathomorphological findings of the cats are summarized in Additional file 2: Table S2.

The liver and kidney samples were stored in polyethylene bags and frosted at -18°C prior to the further analysis. For the pathomorphological investigation, representative tissue samples were fixed in 4% neutral-buffered formaldehyde and subsequently embedded in paraffin. The samples were sectioned at 4 μm, mounted on glass slides and stained with haematoxylin and eosin (H&E).

### Preparation of the samples

The tissue samples were defrosted at room temperature, and 0.5 g of each sample was mixed with 5 ml concentrated nitric acid (65%) in a quartz vessel. The mixture was put into a Teflon® capsule, which was prepared with 5 ml bidistilled water and 2 ml hydrogen peroxide. Subsequently, the Teflon® capsule was placed into a pressure vessel for a wet-chemical digestion in a microwave (MLS GmbH, Leutkirch, Germany). The acid digestion lasted for 1 h, where temperatures up to 200°C were reached. After cooling, the samples were pipetted into graduated flasks that were prepared with bidistilled water. Afterwards, the graduated flasks were filled up with bidistilled water to a total volume of 50 ml.

### Measurement of the elements

The concentrations of Sr, Ba, Cd, Cu, Zn, Mn, Cr, Sb, Se and Pb were measured using ICP-MS (model ELEMENT 1, Finnigan MAT, Bremen, Germany). The validity of the method was verified by the certified NBS SRM biological standard 1577 (bovine liver) (Spex Industries GmbH, Grasbrunn, Germany). For quality assurance, the certified standard was digested and measured in the same way as all the samples, and the results of the analysis are presented in Table 5. For quality control (QC), the solution of a digested horse liver (20 g digested in 20 parts and diluted in 1 l water containing 6% nitric acid) was used. The loop for the measurements was: blank, sample, sample, blank, QC-solution, sample, sample etc. During the entire series, the variation of the elements in the QC-solution was in between 10%. The detection limits for the elements were estimated from the mean of the blank, the standard deviation of the blank and some confidence factor (Table 6).

**Table 5:** Results (expressed in mg/kg) of the analyzed certified reference material NBS SRM biological standard 1577 (bovine liver)

	**Certified value**	**Found**
**Sr**	140 (not certified)	135 ± 9
**Ba**	0.94 (not certified)	0.89 ± 0.06
**Cd**	0.44 ± 0.06	0.48 ± 0.06
**Cu**	158 ± 7	156 ± 8
**Zn**	123 ± 8	120 ± 9
**Mn**	9.9 ± 0.8	9.5 ± 0.8
**Cr**	0.15 (not certified)	0.19 ± 0.06
**Sb**	0.0003 (not certified)	Not detected
**Se**	0.71 ± 0.07	0.69 ± 0.07
**Pb**	0.135 ± 0.015	0.132 ± 0.012

**Table 6:** Limit of detection (μg/kg) and limit of quantification (μg/kg) in the present study

	**Limit of detection**	**Limit of quantification**
**Sr**	0.007	0.021
**Ba**	0.007	0.021
**Cd**	0.056	0.168
**Cu**	0.053	0.159
**Zn**	0.041	0.123
**Mn**	0.077	0.231
**Cr**	0.056	0.168
**Sb**	0.006	0.018
**Se**	0.500	1.500
**Pb**	0.006	0.018

### Statistical methods

The data were analyzed using SPSS 15 (Chicago, USA). As the data were not normally distributed, the non-parametric Mann-Whitney-U-Test was performed for the evaluation of group differences, and *P* < 0.05 was considered as statistically significant. The data are presented in tables as median (minimum-maximum).

## Abbreviations

Ba: Barium; Cd: Cadmium; CKD: Chronic kidney disease; Cr: Chromium; Cu: Copper; DM: Dry matter; H&E: Haematoxylin and eosin; ICP-MS: Inductively coupled plasma mass spectrometry; Mn: Manganese; Pb: Lead; Sb: Antimony; Se: Selenium; Sr: Strontium; Zn: Zinc.

## Competing interests

The authors declare that they have no competing interests.

## Authors’ contributions

NP wrote the manuscript and carried out data analysis. BM carried out sample analysis and participated in data analysis and literature review. ML-W and HS participated in study design and review of the manuscript. RP participated in sample analysis. AB provided information on the pathomorphological findings of the cats and participated in reviewing the manuscript. JZ participated in study design, data analysis and review of the manuscript. All authors have read and approved the final manuscript.

## Supplementary Material

Additional file 1: Table S1 Concentrations (μg/kg) of strontium (Sr), barium (Ba), cadmium (Cd), copper (Cu), zinc (Zn), manganese (Mn), chromium (Cr), antimony (Sb), selenium (Se) and lead (Pb) in the liver and kidneys of cats, depending on the sex and age of the animals^1^. Median (Minimum-Maximum). Click here for file

Additional file 2: Table S2 Sex, age, breed and pathomorphological findings of the cats included in this study. Click here for file
