# Supplementary material for: Liver and kidney concentrations of strontium, barium, cadmium, copper, zinc, manganese, chromium, antimony, selenium and lead in cats
Source: BMC Vet Res. 2014 Jul 17;10:163. doi: 10.1186/1746-6148-10-163 (PMC4108249; doi:10.1186/1746-6148-10-163)
Supplement: Additional file 1: Table S1 — Concentrations (μg/kg) of strontium (Sr), barium (Ba), cadmium (Cd), copper (Cu), zinc (Zn), manganese (Mn), chromium (Cr), antimony (Sb), selenium (Se) and lead (Pb) in the liver and kidneys of cats, depending on the sex and age of the animals1. Median (Minimum-Maximum). [file 1746-6148-10-163-S1.doc]

**Additional file 1: Table S1:** Concentrations (μg/kg) of strontium (Sr), barium (Ba), cadmium (Cd), copper (Cu), zinc (Zn), manganese (Mn), chromium (Cr), antimony (Sb), selenium (Se) and lead (Pb) in the liver and kidneys of cats, depending on the sex and age of the animals^1^. Median (Minimum-Maximum)

|  | **Age** | **Liver** | | | | **Cortex of the kidney** | | | | **Renal medulla** | | | |
| --- | --- | --- | --- | --- | --- | --- | --- | --- | --- | --- | --- | --- | --- |
|  |  | **♂** | | **♀** | | **♂** | | **♀** | | **♂** | | **♀** | |
| **Sr** | **0-1** | 168 | (6.00-2699) | 165 | (39.6-316) | 200 | (21.2-1027) | 157 | (38.7-191) | 162 | (11.2-364) | 188 | (40.8-217) |
|  | **1.5-4** | 47.1 | (0.00-121) | 109 | (71.9-138) | 77.1 | (48.6-80.6) | 61.4 | (54.1-132) | 79.0 | (7.26-99.0) | 59.2 | (56.7-153) |
|  | **4.5-7** | 62.8 | (4.33-121) | 46.8 | (0.00-163) | 75.5 | (11.3-140) | 82.6 | (0.00-251) | 125 | (28.8-222) | 130 | (0.00-625) |
|  | **8-11** | 127 | (60.6-228) | 29.6 | | 154 | (81.4-728) | 42.1 | | 140 | (56.5-207) | 42.7 | |
|  | **12-18** | 126 | (23.0-131) | 58.7 | (12.9-166) | 129 | (53.9-168) | 159 | (37.2-512) | 136 | (106-178) | 178 | (65.7-926) |
| **Ba** | **0-1** | 174 | (0.00-662) | 127 | (82.6-204) | 183 | (0.00-258) | 136 | (107-210) | 180 | (0.00-1049) | 211 | (116-311) |
|  | **1.5-4** | 217 | (192-303) | 211 | (67.5-225) | 239 | (207-387) | 287 | (209-315) | 210 | (190-248) | 232 | (110-262) |
|  | **4.5-7** | 59.6 | (0.00-119) | 148 | (0.00-236) | 70.1 | (0.00-140) | 188 | (0.00-258) | 68.8 | (0.00-138) | 146 | (0.00-245) |
|  | **8-11** | 182 | (75.6-1659) | 0 | | 214 | (151-2628) | 69.0 | | 167 | (79.0-960) | 0 | |
|  | **12-18** | 75.3 | (0.00-130) | 98.9 | (0.00-215) | 108 | (0.00-191) | 145 | (0.00-471) | 75.4 | (0.00-136) | 93.4 | (0.00-405) |
| **Cd** | **0-1** | 109 | (7.30-206) | 135 | (31.9-264) | 114 | (4.13-209) | 148 | (7.50-231) | 105 | (3.41-225) | 119 | (2.74-194) |
|  | **1.5-4** | 86.5 | (26.5-196) | 209 | (146-226) | 98.7 | (41.1-116)^a^ | 208 | (141-347)^b^ | 46.9 | (3.10-79.3)^a^ | 133 | (104-179)^b^ |
|  | **4.5-7** | 160 | (90.3-229) | 109 | (50.4-803) | 222 | (80.0-363) | 144 | (51.8-467) | 94.7 | (42.9-146) | 72.5 | (3.98-525) |
|  | **8-11** | 198 | (86.4-504) | 159 | | 167 | (106-444) | 350 | | 91.8 | (38.0-160) | 88.9 | |
|  | **12-18** | 503 | (294-583)^a^ | 165 | (62.5-271)^b^ | 292 | (151-2920) | 414 | (214-872) | 137 | (66.6-1349) | 137 | (43.9-210) |
| **Cu** | **0-1** | 15004 | (4237-98689) | 64387 | (14419-95029) | 3419 | (2042-15656) | 3175 | (1637-5575) | 2354 | (1645-5939) | 2867 | (1253-4674) |
|  | **1.5-4** | 9843 | (3750-16489)^a^ | 20747 | (16516-86070)^b^ | 2103 | (1814-3291) | 2457 | (2451-2746) | 1883 | (1094-2792) | 2291 | (2227-2754) |
|  | **4.5-7** | 41446 | (36547-46345) | 26830 | (2701-72700) | 2458 | (2445-2470) | 2426 | (1119-5651) | 2148 | (2127-2169) | 1385 | (485-2761) |
|  | **8-11** | 31682 | (8876-91443) | 38696 | | 3077 | (1731-3598) | 2382 | | 1594 | (1217-2594) | 1814 | |
|  | **12-18** | 20842 | (5236-21889) | 24729 | (12567-48483) | 2708 | (1255-2821) | 2644 | (1290-3167) | 1879 | (1174-2531) | 1677 | (895-2348) |
| **Zn** | **0-1** | 28606 | (16608-46017) | 49355 | (18531-69164) | 15480 | (10816-24389) | 20997 | (8280-33896) | 13320 | (9249-18405) | 15316 | (5005-17276) |
|  | **1.5-4** | 24619 | (14353-33056) | 27610 | (23952-35020) | 13796 | (10672-17993) | 13536 | (10957-13922) | 8318 | (5655-15193) | 10520 | (10230-11293) |
|  | **4.5-7** | 28439 | (20046-36833) | 19960 | (14975-41960) | 13370 | (8446-18295) | 9710 | (8431-35290) | 10761 | (5810-15712) | 6924 | (4687-14664) |
|  | **8-11** | 35836 | (15576-89115) | 32389 | | 15855 | (11051-19305) | 11818 | | 9422 | (6036-11938) | 7889 | |
|  | **12-18** | 40883 | (8825-60234) | 30675 | (13379-42989) | 14065 | (5839-18508) | 10360 | (5872-27943) | 10174 | (5622-14345) | 8433 | (3778-14762)1s), n=1s), n=81 years), n=6 |
| **Mn** | **0-1** | 3982 | (2155-6162) | 5379 | (1311-13098) | 1365 | (910-2592) | 1841 | (786-2077) | 897 | (348-1451) | 1040 | (380-1692) |
|  | **1.5-4** | 3076 | (2111-4144) | 3032 | (2274-3218) | 1305 | (836-1969) | 1088 | (1000-1308) | 712 | (338-1651) | 774 | (769-1104) |
|  | **4.5-7** | 2883 | (2661-3105) | 2405 | (1485-7073) | 1366 | (1077-1654) | 908 | (502-1382) | 663 | (600-726) | 697 | (233-916) |
|  | **8-11** | 2682 | (1767-5052) | 3108 | | 1082 | (738-1164) | 1312 | | 561 | (417-668) | 733 | |
|  | **12-18** | 3369 | (1403-4248) | 2555 | (2055-3731) | 1060 | (511-1108) | 1002 | (733-1452) | 541 | (375-869) | 633 | (358-714) |
| **Cr** | **0-1** | 294 | (29.2-347) | 266 | (9.36-368) | 284 | (84.1-327) | 263 | (10.7-321) | 243 | (100-441) | 275 | (16.0-426) |
|  | **1.5-4** | 170 | (55.0-280) | 98.9 | (57.2-345) | 167 | (57.1-172) | 87.2 | (50.7-291) | 138 | (75.1-243) | 214 | (53.3-337) |
|  | **4.5-7** | 249 | (197-302) | 196 | (13.6-337) | 235 | (186-285) | 135 | (32.4-314) | 223 | (133-313) | 135 | (13.9-250) |
|  | **8-11** | 248 | (60.8-354) | 215 | | 237 | (71.5-376) | 114 | | 149 | (21.4-296) | 135 | |
|  | **12-18** | 259 | (190-296) | 279 | (33.8-347) | 290 | (128-342) | 203 | (123-384) | 324 | (132-332) | 215 | (90.0-436) |
| **Sb** | **0-1** | 169 | (0.00-191) | 186 | (0.00-190) | 159 | (0.00-236) | 180 | (0.00-185) | 163 | (0.00-377) | 182 | (0.00-243) |
|  | **1.5-4** | 18.5 | (0.00-24.2) | 51.6 | (20.8-191) | 11.4 | (0.00-13.1) | 13.1 | (10.9-180) | 11.2 | (0.00-12.7) | 12.2 | (10.7-180) |
|  | **4.5-7** | 178 | (155-201) | 78.7 | (0.00-197) | 159 | (133-185) | 57.7 | (0.00-186) | 161 | (133-188) | 54.8 | (0.00-217) |
|  | **8-11** | 25.6 | (0.00-194) | 159 | | 11.3 | (0.00-187) | 115 | | 11.8 | (0.00-184) | 118 | |
|  | **12-18** | 185 | (137-190) | 132 | (0.00-216) | 171 | (133-186) | 128 | (0.00-178) | 175 | (128-189) | 126 | (0.00-168) |
| **Se** | **0-1** | 570 | (73.3-844) | 662 | (434-696) | 1168 | (257-1898) | 1159 | (929-1565) | 829 | (123-1316) | 814 | (681-1240) |
|  | **1.5-4** | 301 | (215-393)^a^ | 495 | (396-1792)^b^ | 595 | (68.4-900) | 1016 | (822-3429) | 407 | (231-634) | 587 | (443-3429) |
|  | **4.5-7** | 616 | (292-940) | 276 | (179-1167) | 1138 | (589-1687) | 641 | (405-1068) | 785 | (320-1251) | 283 | (90.1-1912) |
|  | **8-11** | 601 | (276-693) | 258 | | 1094 | (643-2145) | 408 | | 481 | (339-1315) | 265 | |
|  | **12-18** | 957 | (174-1163) | 441 | (255-1421) | 2422 | (321-2699) | 1126 | (346-2178) | 1844 | (223-2044) | 786 | (226-1619) |
| **Pb** | **0-1** | 255 | (16.4-442) | 222 | (0.82-275) | 248 | (6.67-289) | 211 | (1.63-216) | 219 | (95.3-300) | 210 | (0.63-278) |
|  | **1.5-4** | 42.9 | (2.40-56.2) | 35.7 | (24.7-215) | 10.8 | (1.35-30.1) | 27.0 | (24.2-213) | 8.30 | (0.44-26.5) | 9.40 | (7.19-213) |
|  | **4.5-7** | 147 | (82.9-212) | 50.9 | (2.40-101) | 146 | (82.4-209) | 62.4 | (0.39-229) | 149 | (83.2-215) | 54.9 | (0.00-329) |
|  | **8-11** | 66.2 | (2.56-264) | 337 | | 134 | (28.5-214) | 140 | | 17.6 | (2.47-202) | 107 | |
|  | **12-18** | 215 | (92.9-294) | 92.9 | (1.51-243) | 206 | (87.1-217) | 82.9 | (27.7-267) | 201 | (81.0-216) | 80.8 | (19.9-335) |

^1^♂: n=6 (0-1 years), n=5 (1.5-4 years), n=2 (4.5-7 years), n=6 (8-11 years), n=3 (12-18 years); ♀: n=4 (0-1 years), n=3 (1.5-4 years), n=10 (4.5-7 years), n=1 (8-11 years), n=7 (12-18 years)

Group comparisons were separately calculated for each element and each tissue. Differences (*P* < 0.05) within a row are marked with different superscript letters
