# Supplementary material for: Liver and kidney concentrations of strontium, barium, cadmium, copper, zinc, manganese, chromium, antimony, selenium and lead in cats
Source: BMC Vet Res. 2014 Jul 17;10:163. doi: 10.1186/1746-6148-10-163 (PMC4108249; doi:10.1186/1746-6148-10-163)
Supplement: Additional file 2: Table S2 — Sex, age, breed and pathomorphological findings of the cats included in this study. [file 1746-6148-10-163-S2.doc]

**Additional file 2: Table S2:** Sex, age, breed and pathomorphological findings of the cats included in this study

| **No.** | **Sex** | **Age** | **Breed** | **Pathomorphological findings** |
| --- | --- | --- | --- | --- |
| 1 | ♀ | 6 years | European shorthair | hepatic lipidosis |
| 2 | ♂ | 10 years | European shorthair | chronic interstitial nephritis; renal fibrosis and glomerulosclerosis |
| 3 | ♀ | 6 years | Persian | chronic interstitial nephritis; polycystic kidney disease |
| 4 | ♂ | 4 years | European shorthair | cardiomyopathy; glomerulosclerosis |
| 5 | ♀ | 16 years | European shorthair | glomerulosclerosis; chronic interstitial nephritis |
| 6 | ♂ | 3 years | European shorthair | lesions indicative for Parvovirus infection |
| 7 | ♂ | 4 years | Persian crossbreed | lesions indicative for Parvovirus infection |
| 8 | ♂ | 10 years | European shorthair | spindle cell tumor (nasal cavity, brain); chronic interstitial nephritis |
| 9 | ♂ | 3 years | European shorthair | lesions indicative for Parvovirus infection |
| 10 | ♀ | 5 years | European shorthair | chronic interstitial nephritis; liver cirrhosis |
| 11 | ♀ | 2 years | European shorthair | lesions indicative for Parvovirus infection |
| 12 | ♂ | 1 year | European shorthair | lesions indicative for Parvovirus infection |
| 13 | ♀ | 2 years | European shorthair | cardiovascular failure (unknown reason) |
| 14 | ♀ | 13 years | European shorthair | adenocarcinoma in the lungs; chronic interstitial nephritis; cardiomyopathy |
| 15 | ♂ | 8 years | Maine Coon crossbreed | lesions indicative for feline asthma; chronic interstitial nephritis; purulent and lymphoplasmacellular meningitis |
| 16 | ♀ | 4.5 years | European shorthair | lesions indicative for Feline infectious peritonitis virus infection; chronic interstitial nephritis; chronic hepatitis |
| 17 | ♀ | 2 months | European shorthair | lesions indicative for virus infection (cat flu complex) |
| 18 | ♂ | 3 months | European shorthair | atresia coli et recti |
| 19 | ♂ | 8.5 years | European shorthair | foreign body (small intestine) |
| 20 | ♂ | 7 years | European shorthair | lesions indicative for Parvovirus and bacterial infection; chronic interstitial nephritis; chronic degeneration of the liver |
| 21 | ♂ | 3 months | European shorthair | hepatocellular necrosis |
| 22 | ♂ | 1 year | European shorthair | necrotizing pneumonia (indicative for infection with agents of the cat flu complex) |
| 23 | ♂ | 14 years | European shorthair | adenocarcinoma of the lungs and of the pancreas; chronic interstitial nephritis; islet amyloidosis |
| 24 | ♀ | 3 years | Maine Coon | cardiomyopathy |
| 25 | ♀ | 16 years | European shorthair | cardiomyopathy; adenocarcinoma of the mammary gland and metastases in the lungs; mast cell tumor |
| 26 | ♀ | 14 years | European shorthair | squamous cell carcinoma in the region of the glottis; chronic interstitial nephritis |
| 27 | ♀ | 3 months | British shorthair | fibrosing pancreatitis |
| 28 | ♂ | 15 years | European shorthair | meningioma (consecutive compression of the cerebral cortex) |
| 29 | ♂ | 8 years | European shorthair | cardiomyopathy; chronic interstitial nephritis |
| 30 | ♀ | 3 months | Turkish angora | anemia; hepatocellular necrosis; cardiomyopathy; auto erythrophagocytic syndrome |
| 31 | ♀ | 18 years | European shorthair | thyroid adenoma; chronic interstitial nephritis |
| 32 | ♀ | 7 years | European shorthair | thymoma; degeneration of hepatocytes and liver bridging fibrosis |
| 33 | ♀ | 12 years | Maine Coon crossbreed | fibrosis of the kidney; liver fibrosis; cardiomyopathy |
| 34 | ♀ | 13 years | European shorthair | myeloma (medullar and extramedullar); hepatocellular necrosis |
| 35 | ♂ | 14 years | European shorthair | chronic interstitial nephritis |
| 36 | ♂ | 6 years | European shorthair | chronic interstitial nephritis; acute hepatitis |
| 37 | ♀ | 6 years | Persian Crossbreed | adenocarcinoma of the stomach |
| 38 | ♀ | 6 years | European shorthair | chronic interstitial nephritis |
| 39 | ♂ | 2 months | European shorthair | heptocellular necrosis; lesions indicative for Parvovirus infection |
| 40 | ♂ | 1.5 years | European shorthair | heptocellular necrosis; foreign body in the stomach |
| 41 | ♀ | 7 years | Persian | purulent-necrotizing hepatitis; chronic interstitial nephritis |
| 42 | ♀ | 5 years | European shorthair | chronic interstitial nephritis |
| 43 | ♂ | 2 months | European shorthair | lesions indicative for Parvovirus infection |
| 44 | ♀ | 10 years | European shorthair | chronic interstitial nephritis; heptocellular necrosis; lymphoma |
| 45 | ♂ | 9 years | European shorthair | cardiac insufficiency |
| 46 | ♀ | 7 years | European shorthair | hepatocellular necrosis; chronic interstitial nephritis, adenocarcinoma of the lungs; lesions indicative for Feline parvovirus infection |
| 47 | ♀ | 1 year | Maine Coon | cardiomyopathy; hydrothorax |

* Cardiomyopathy: including primary (idiopathic) and secondary (etiologic) cardiomyopathy
